# Compliance to D2 lymphadenectomy in laparoscopic gastrectomy

**DOI:** 10.1007/s13304-018-0553-1

**Published:** 2018-06-20

**Authors:** Wietse J. Eshuis, Mark I. van Berge Henegouwen, Werner A. Draaisma, Suzanne S. Gisbertz

**Affiliations:** 1 Department of Surgery, Academic Medical Center, Meibergdreef 9, 1105 AZ Amsterdam, The Netherlands; 2 Present Address: Department of Surgery, Jeroen Bosch Ziekenhuis, ‘s Hertogenbosch, The Netherlands

**Keywords:** Gastric cancer, Minimally invasive surgery, Lymphadenectomy

## Abstract

The objective of this study is to describe the compliance to D2 lymphadenectomy in laparoscopic gastrectomy. Radical partial or total gastrectomy with modified D2 lymphadenectomy is the standard of care for locally advanced gastric cancer. It is unclear whether compliance to D2 lymphadenectomy in laparoscopy is comparable to that in open surgery. A review of the literature was performed and results are described in a descriptive review. Available randomized trials are mostly performed for early gastric cancer, for which formal D2 lymphadenectomy is usually not required. Most trials report no differences in number of retrieved lymph nodes between open and laparoscopic gastrectomy. Only one trial used adherence to D2 lymphadenectomy as primary outcome parameter, and found no difference between laparoscopic and open gastrectomy. Results from randomized trials in advanced gastric cancer are awaited. In the meantime, the laparoscopic approach can be used in experienced centers.

## Introduction

Gastric cancer is the fifth most frequently diagnosed cancer worldwide [1]. With an estimated number of 723,100 deaths annually, it ranks third in the list of most deadly cancers [1]. Successful treatment of gastric cancer is a multidisciplinary effort, of which a high-quality surgical resection is the mainstay. The standard of surgical resection for resectable gastric cancer is a radical gastrectomy including a lymphadenectomy.

**Fig. 1:**
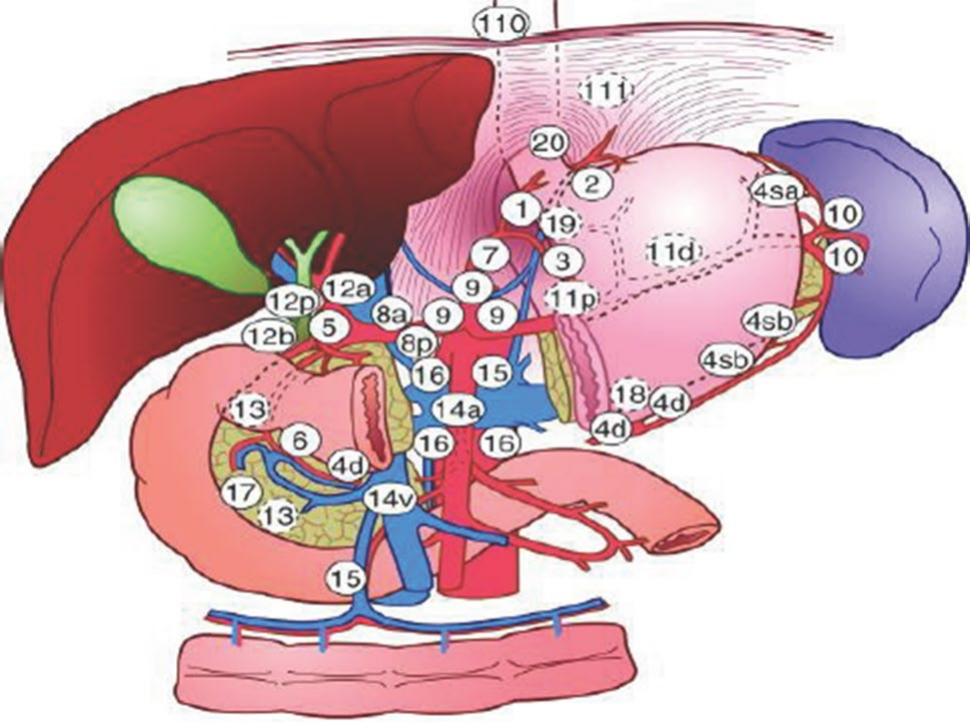
Lymph-node stations according to the Japanese gastric cancer guidelines

The extent of lymphadenectomy is described according to the guidelines of the Japanese Gastric Cancer Association [2]. Lymph-node stations are numbered systematically, with lowest numbers corresponding to the direct perigastric N1 stations along the lesser (1, 3, and 5) and greater curvature (2, 4, and 6) (see Fig. 1). Lymph-node stations 7–12 are grouped as N2, and correspond to the stations around the left gastric artery (7), common hepatic artery (8), celiac trunk (9), splenic artery (10 and 11), and hepatoduodenal ligament (12). Stations 13–15 (behind the pancreas and along the superior mesenteric and middle colic vessels) are considered N3, and stations 16 (para-aortic) and higher are considered N4.

D1 lymphadenectomy consists of removal of D1 lymph-node stations, while D2 lymphadenectomy consists of removal of D1 and D2 nodes (see Table 1).

**Table 1:** Lymph-node stations to be removed for D1 and D2 lymphadenectomy in total and distal gastrectomy

Resection	Lymph-node stations to be removed	
Total gastrectomy		
D1	1–7	
D1+	D1+ 8a, 9, 11p	
D2	D1+ 8a, 9, 10, 11p, 11d, 12a	
Distal gastrectomy		
D1	1, 3, 4sb, 4d, 5, 6, 7	
D1+	D1+ 8a, 9	
D2	D1+ 8a, 9, 11p, 12a	

For early gastric cancer with no nodal involvement, a D1 or D1+ lymphadenectomy is indicated [2]. In advanced gastric cancer (T2-4a and/or N+), a D2 lymphadenectomy is associated with a better survival [3, 4]. Originally, an official D2 lymphadenectomy included removal of the pancreatic tail and spleen. However, since no survival benefit of these organ resections was demonstrated, and a higher postoperative morbidity was seen, a ‘modified’ D2 lymphadenectomy has been developed, with preservation of the pancreas and spleen, but including the splenic hilar lymph nodes. The modified D2 lymphadenectomy has recently been proven to be non-inferior with regard to overall survival [5]. With the advent of the ‘modified’ D2 lymphadenectomy and its associated decrease in morbidity, most guidelines now advise a D2 lymphadenectomy in these patients [2, 6]. Splenectomy is to be preserved for cases with direct tumor invasion of the spleen or tumor location in the greater curvature of the upper stomach.

D2 lymphadenectomy is a technically more challenging procedure than D1 lymphadenectomy. Even in open surgery, compliance to D2 lymphadenectomy is demanding. Overall non-compliance in the Dutch Gastric Cancer Trial was more than 80% in the D2 group [7]. Like in other gastrointestinal malignancies, laparoscopic surgery has been adopted widely among gastric cancer surgeons. It has been shown to be safe and technically feasible in gastrectomy, with an additional benefit of less wound complications compared to open surgery [8]. However, due to the increased difficulty of laparoscopic dissection, doubts have been raised whether laparoscopic surgery is also safe from an oncologic point of view, especially in advanced gastric cancer, where an adequate D2 lymphadenectomy is required. In this review, we will discuss the compliance to D2 lymphadenectomy in laparoscopic gastrectomy. We will review the available literature on lymphadenectomy in laparoscopic gastrectomy, with a distinction between Asian and Western literature, discuss ongoing trials, and elaborate on the pros and cons of laparoscopic gastrectomy.

## Evidence from randomized trials

Several randomized trials have compared laparoscopic with open gastrectomy, most of which report the number of harvested lymph nodes. Here, an overview of trials is presented that compare laparoscopic (or laparoscopy-assisted) with open gastrectomy for cancer and provide outcomes with regard to lymph-node dissection (see also Table 2).

**Table 2:** Studies randomizing between open and laparoscopic gastrectomy, which provide outcomes related to lymph-node dissection

Study	No. of subjects	Early/advanced gastric cancer	Type(s) of resection performed	Type(s) of lymphadenectomy performed	No. of harvested lymph nodes (lap versus open)*	Additional data lymphadenectomy (lap versus open)	Remarks
Kitano et al. (2002) [9]	28	Early gastric cancer	Distal gastrectomy	Only perigastric lymph-node dissection (D1)	20.2 vs 24.9 (NS)	–	All operations by 1 surgeon
Hayashi et al. (2005) [10]	28	Early gastric cancer	Distal gastrectomy	D2, only station 12a not clearly described	28 vs 27 (NS)	–	All laparoscopic operations by 1 surgeon
Lee et al. (2005) [11]	47	Early gastric cancer	Distal gastrectomy	D2; open group included also stations 12p and 13	31.8 vs 38.1 (NS)	–	–
Huscher et al. (2005) [12]	59	All stages	Distal gastrectomy	D1 or extended D2 (including cholecystectomy + stations 12b, 12p, 13, 17)	30.0 vs 33.4 (NS)	–	The only Western trial
Cai et al. (2011) [13]	123	All stages	Proximal, distal or total gastrectomy	D2 including station 14v	23.0 vs 22.9 (NS)	–	Only 96 patients with advanced gastric cancer included in analysis
Chen Hu et al. (2012) [14]	88	Only N0 stages	Distal gastrectomy	D1 or D2	17.6/18.9 vs 19.1/18.8 (NS)	–	Also randomized between fast-track/conventional care
Sakuramoto et al. (2013) [15]	64	Early gastric cancer	Distal gastrectomy	D2, only station 12a not clearly described	31.6 vs 33.8 (NS)	–	–
Takiguchi et al. (2013) [16]	40	Early gastric cancer	Distal gastrectomy	D1 (mostly) or D2	Median 33 vs 32 (NS)	–	–
Aoyama et al. (2014) [17]	26	Early gastric cancer	Distal gastrectomy	D1 or D2	Median 40.5 vs 43 (NS)	–	Main outcomes: surgical stress and nutritional status
Cui et al. (2015) [18]	296	Advanced gastric cancer	Proximal, distal or total gastrectomy	D2	29.3 vs 30.1 (NS)	No differences when stratified for type of resection	Conversions were not analyzed ITT
Kim et al. (2016) [19]	1415	Early gastric cancer	Distal gastrectomy	D1 + or D2	40.5 vs 43.7 (*P* < 0.001)	More D2 in open group (64% vs 56%)	First multicenter trial, strict quality control
Hu et al. (2016) [21]	1056	Advanced gastric cancer	Distal gastrectomy	D2	36.1 vs 36.9 (NS)	Compliance to D2 99.4% vs 99.6%	Multicenter trial, strict quality control
Katai et al. (2016)	921	Early gastric cancer	Distal gastrectomy	D1, D1 + or D2	Median 39 vs 39 (NS)	No difference in distribution of extent of nodal dissection	Multicenter trial, strict quality control
Park et al. (2017) [23]	204	Advanced gastric cancer	Distal gastrectomy	D2	37.0 vs 39.7 (NS)	Non-compliance to D2 47% vs 43% (NS), but 52% vs 25% (*P* 0.043) in stage III subgroup	Multicenter trial, strict quality control. Main outcome: non-compliance to D2

* Mean, unless stated otherwise

The first randomized trial comparing open with laparoscopic gastrectomy included only early gastric cancers for which a distal gastrectomy was performed [9]. All operations were carried out by one surgeon and 14 patients were included in each group. The mean number of harvested lymph nodes did not differ significantly between the laparoscopic and open groups (20.2 versus 24.9, respectively). However, in this trial, only a perigastric lymph-node dissection (D1) was performed.

The trial from Hayashi et al. also described two groups of 14 patients each, who underwent open or laparoscopy-assisted distal gastrectomy with extraperigastric lymph-node dissection for early gastric cancer [10]. All laparoscopy-assisted operations were carried out by the same surgeon. In the ‘Methods’ section, a D2 lymphadenectomy seems to be described, although station 12a is not clearly included in the dissection. Mean number of harvested lymph nodes was 28 (laparoscopy-assisted) versus 27 (open) (not significant).

In the same year, a randomized trial from Korea was published [11], which included 47 distal gastrectomies for early gastric cancer. In this trial, a D2 lymphadenectomy was mandatory, but apart from that, in the open group, even more lymph-node stations were resected, namely the posterior side of the hepatoduodenal ligament (12p) and retropancreatic nodes (13). This difference in approach precludes an honest comparison between the groups, although no difference in number of harvested lymph nodes was found (mean 31.8 in the laparoscopic group, versus 38.1 in the open group, not significant).

The only Western trial on laparoscopic versus open gastrectomy was also published in 2005 [12]. It was performed in Italy and included 59 patients who underwent a distal gastrectomy. All patients were operated by one surgeon. An extensive D2 lymphadenectomy is described in the ‘Methods’ section, including a cholecystectomy and dissection of lymph nodes of group 17 (along pancreaticoduodenal artery), 13 (retropancreatic), and 12p and 12b ligamental nodes. All stages of gastric cancer were included; this has also led to the inclusion of early gastric cancers, with only a D1 lymphadenectomy being performed in 9/29 patients in the open group and 9/30 patients in the laparoscopic group. No significant difference was found in the mean number of resected lymph nodes (33.4 in the open group compared to 30.0 in the laparoscopic group).

The first trial to include other resections than distal gastrectomy was published in 2011 by Cai and coworkers [13]. 123 patients were included, who underwent proximal, distal, and total gastrectomies. Only a subgroup of 96 patients with advanced cancer was analyzed. All patients underwent D2 lymphadenectomy including station 14v. No difference in mean number of harvested lymph nodes was found: 23.0 in the laparoscopic group versus 22.9 in the open group.

Chen Hu et al. published an RCT on laparoscopic versus open distal gastrectomies, in which patients were also randomized between fast-track and conventional care [14]. Only stages without lymph-node metastasis were included, and both D1 and D2 lymphadenectomies were performed. No difference in mean lymph-node harvest was found between the four treatment groups (17.6 laparoscopy with fast-track, 18.9 laparoscopy with conventional care, 19.1 open with fast-track, and 18.8 open with the conventional care).

The next trial that was published also included only 64 distal gastrectomies for early gastric cancers [15]. The lymphadenectomy which the authors describe comes down to a D2 lymphadenectomy minus station 12a. Mean number of harvested lymph nodes was 31.6 in the laparoscopic group versus 33.8 in the open group (not significant).

After that, two small trials from Japan were published. Takiguchi et al. randomized 40 patients with early gastric cancer to open or laparoscopic distal gastrectomy, all performed by one surgeon [16]. Although a D2 lymphadenectomy minus station 12a is described in their methods, they state that only one patient in each group underwent a D2 lymphadenectomy; the rest all D1. Median number of resected lymph nodes was 33 (laparoscopy) versus 32 (open, not significant). The trial by Aoyama et al., who randomized 26 patients with stage I gastric cancer to laparoscopic or open distal gastrectomy, investigated surgical stress and nutritional status but also reported lymph-node harvest [17]. Both D1 and D2 lymphadenectomies were performed; median number of harvested lymph nodes was 40.5 in the laparoscopic group versus 43 in the open group (not significant).

From 2015 onwards, trials became larger, with more included patients and later also more multicenter trials. Cui and coworkers randomized almost 300 patients with advanced gastric cancers to open or laparoscopic proximal, distal, or total gastrectomy [18]. D2 lymphadenectomy was required. Conversions to open surgery were excluded from analysis. Mean number of harvested lymph nodes was not significantly different (29.3 versus 30.1 in the laparoscopic and open groups, respectively). All patients had at least 15 lymph nodes resected. When stratified according to type of resection, there were still no differences.

The KLASS 01 trial was the first multicenter trial that was published, recruiting 1415 patients scheduled for distal gastrectomy for early gastric cancer in 12 Korean hospitals [19]. Lymphadenectomies were D1+ or D2. In the laparoscopic group, there were less wound complications, longer operation times, and: less lymph nodes resected, 40.5 versus 43.7, *P* < 0.001. More D2 lymphadenectomies were performed in the open group, although cancer stages were equally distributed among both groups. Three open and four laparoscopically operated patients had less than 15 lymph nodes resected, respectively. The trial was subjugated to a strict quality control: only large-volume centers could participate (> 80 gastric resections per year), and there were video and photo-review procedures to ensure the quality of the operations [20]. The authors conclude that D2 lymphadenectomy is regarded as procedure of choice in open surgery, but, that in laparoscopic gastrectomy, D1+ lymphadenectomy is also accepted, and that this difference in perception might account for the difference in number of resected lymph nodes and percentage of D2 lymphadenectomy.

Not long after, the multicenter trial by Hu et al. was published [21]. 1056 patients with advanced gastric cancer were randomized in 14 Chinese institutions between open and laparoscopic distal gastrectomy. A D2 lymphadenectomy was required. This trial also adhered to strict quality control measures, including the requirement of photographic evidence of surgical lymph-node dissection fields, resection margin, and incision. Adherence to D2 lymphadenectomy was 99.4 and 99.6% in the laparoscopic and open groups, with respective mean numbers of harvested lymph nodes of 36.1 versus 36.9 (not significantly different).

Katai and coworkers included 921 patients with stage IA or IB gastric cancer scheduled for distal gastrectomy, in 33 hospitals in Japan [22]. Lymphadenectomy was either D1/D1+ (for stage IA) or D2 (for stage IB, approximately 25%). Participating surgeons were required to supply photo- and video-graphic evidence of the laparoscopic procedures. Median number of harvested lymph nodes was 39 in both groups. In addition, there was no difference in the distribution of the extent of nodal dissection between the groups.

The most recent multicenter trial, the COACT 1001, was performed in Korea by Park et al. [23]. Only patients with advanced gastric cancer who underwent distal gastrectomy were included. This trial also used strict quality control measures. Only surgeons who had performed at least 30 laparoscopic distal gastrectomies could participate. Procedures were taught by use of video seminars of ten unedited procedures. This is the only trial that used compliance to D2 lymphadenectomy as its primary outcome parameter. Non-compliance was defined as more than one empty lymph-node station. Further quality control was performed by reviewing unedited laparoscopic videos and, in case of open procedures, photo documentation, with help of a checklist [24]. Non-compliance rate was 47.0% in the laparoscopic group, compared to 43.2% in the open group (not statistically significant). Subgroup analysis revealed no difference in non-compliance for stage I and stage II disease, but, for stage III disease, a higher non-compliance rate was found in the laparoscopic group (52 versus 25%, *P* = 0.043). There was no difference in number of resected lymph nodes, 37.0 (laparoscopic group) versus 39.7 (open group).

In summary, most of the trials on laparoscopic versus open gastrectomy included only distal gastrectomies for early gastric cancer, and formal D2 lymphadenectomy was not required in most trials. In none of the trials, patients were treated with neoadjuvant chemotherapy. Most trials revealed less wound complications, less blood loss, a longer operation time, and a shorter hospital stay in the laparoscopic group. There was only one trial that reported a significant difference in lymph-node harvest: the KLASS 01 trial [19]. However, this trial included D1+ lymphadenectomies; furthermore, one could argue the clinical relevance of the difference between 40.5 and 43.7 harvested lymph nodes. The only trial that reported compliance to D2 lymphadenectomy as primary outcome measure reported no difference between the laparoscopic and the open approach [23]. However, in the subgroup of patients with stage III disease (53 patients in total), compliance was lower in the laparoscopic group. The authors stated that this may have been caused by increased difficulty of laparoscopic lymphadenectomy, or a more aggressive approach towards lymphadenectomy in open surgery. However, this trial was not powered for stage III disease, and disease-free survival was not significantly affected, so confirmation of these findings in randomized trials is still awaited.

In line with the evidence from randomized trials, a Cochrane review on laparoscopic versus open gastrectomy for gastric cancer that was published in 2016 found, with only ‘very low quality of evidence’, that there was no significant difference in the number of harvested lymph nodes between the laparoscopic and open groups (MD 0.63, 95% CI 1.51–0.25) [8]. This analysis used a fixed-effects model and included 9 trials with 472 patients in total. At the time of the analysis, the results of the recently published large-volume multicenter trials were not yet available.

## Ongoing trials

In conclusion, more evidence is needed, especially in the following patient categories: patients with advanced gastric cancers, patients undergoing total gastrectomies, patients after neoadjuvant chemotherapy, and, last but not least, patients from western countries. Some of the randomized trials that are, therefore, highly anticipated are discussed shortly here, and summarized in Table 3.

**Table 3:** Ongoing multicenter randomized trials comparing laparoscopic with open gastrectomy, which are of interest with regard to D2 lymphadenectomy

Study	Country of origin	Early/advanced gastric cancer	Type(s) of resection	Type(s) of lymphadenectomy performed
KLASS-02	South Korea	Advanced gastric cancer	Distal gastrectomy	D2
KLASS-03	South Korea	Early gastric cancer	Total gastrectomy	D2
JLSSG 0901	Japan	Advanced gastric cancer	Distal gastrectomy	D2
CLASS-01	China	Advanced gastric cancer	Subtotal gastrectomy	D2
LOGICA	Netherlands	All resectable cancer stages	Total and distal gastrectomy	D2
STOMACH	Netherlands	All resectable cancer stages	Total gastrectomy	D2

- KLASS-02 trial (NCT01456598): a Korean multicenter trial that aims to include 1050 patients with locally advanced gastric cancers and no or limited lymph-node metastasis, who are scheduled to undergo distal gastrectomy with D2 lymphadenectomy [25]. Main outcome parameter is 3-year disease-free survival. The initiators hope to show the efficacy of laparoscopic D2 lymphadenectomy, as compared to open. Potential participating centers must submit six unedited laparoscopic and open gastrectomy videos before being allowed to include patients [26].
- KLASS-03 trial (NCT01584336): another Korean multicenter initiative, that will compare laparoscopic with open total gastrectomy in stage I gastric cancer patients. D2 lymphadenectomy is required. Primary outcome measures are morbidity and mortality.
- JLSSG 0901 RCT: after a successful phase II trial, in which the feasibility of laparoscopic distal gastrectomy for advanced gastric cancers was demonstrated, a phase III trial has now been started in Japan [27, 28]. This multicenter trial will include patients with advanced gastric cancer, who will undergo distal gastrectomy with D2 lymphadenectomy.
- CLASS-01 trial (NCT01609309): this Chinese multicenter study will randomize patients with advanced gastric cancers between laparoscopic and open distal gastrectomy. A D2 lymphadenectomy is required; main outcome parameter is 3-year disease-free survival.
- LOGICA trial (NCT02248519): this Dutch multicenter trial will randomize 210 patients with resectable gastric cancer to open or laparoscopic gastrectomy [29]. Neoadjuvant chemotherapy will be given in accordance with Dutch guidelines. Both total and distal gastrectomies are included. Main outcome parameter is length of hospital stay.
- STOMACH trial (NCT02130726): this is a multinational, multicenter randomized trial that was initiated in the Netherlands [30]. The study will include 168 patients with gastric cancer, scheduled for total gastrectomy, after neoadjuvant chemotherapy. Primary outcome measure is the quality of oncological resection, a composite endpoint consisting of radicality of surgery and number of retrieved lymph nodes. Furthermore, results for dissection of separate lymph-node stations will be analyzed. This trial will answer questions about feasibility of laparoscopic D2 lymphadenectomy after chemotherapy and laparoscopic total gastrectomy, and will do so in an entirely Western population.

## Discussion

In summary, data from randomized studies are insufficient to adequately provide an answer to the question whether D2 lymphadenectomy is of the same quality in laparoscopic gastrectomy as in open gastrectomy. Nonetheless, in the Netherlands, the laparoscopic approach has gained popularity in recent years. Apparently, this has not led to a decrease in lymph-node harvest: the study by Brenkman et al. describes the early results after the introduction of minimally invasive gastrectomies in the Netherlands [31]. It is a population-based cohort study that included all patients who underwent gastrectomy for adenocarcinoma in a 4-year time span and were registered in the national Upper GI Cancer Audit. Propensity score matching was used to create comparable groups of laparoscopic and open gastrectomies. Approximately two-thirds of patients underwent some form of neoadjuvant treatment. In the propensity score matched cohort (442 patients in each group), an equal lymph-node harvest was found in both groups (median 21 versus 20, not significantly different). The percentage of patients with 15 or more lymph nodes resected was 79% in the laparoscopic group versus 74% in the open group (*P *= 0.094). In the Academic Medical Center, laparoscopic gastrectomy has gained popularity in recent years, with open surgery since 2013 being reserved for cases with previous upper abdominal surgery, or LOGICA or STOMACH trial participants. In our Western population, with mostly advanced gastric cancers, and approximately two-thirds of patients receiving neoadjuvant chemotherapy, a mean total lymph-node harvest of 27.8 in laparoscopic surgery in the years 2013–2016 has been achieved, compared with 22.6 in open surgery in the years 2011–2012 (*to be published*). These data suggest that also after neoadjuvant chemotherapy, lymphadenectomy is as adequate in laparoscopic surgery in terms of harvested lymph nodes in total as it is in open surgery; however, they must be interpreted with caution, due to the risk of selection bias.

Total lymph-node yield is only a surrogate marker for adequate lymphadenectomy. Data regarding results of separate lymph-node station dissection are not available from randomized trials. Several non-randomized studies try to describe which lymph-node stations are the most difficult to dissect laparoscopically, and some authors have described results of laparoscopic lymphadenectomy separately per station. Miura and coworkers found a significantly lower lymph-node harvest in laparoscopically dissected stations 4, 6, 9, and 11 compared to open surgery [32]. Kawamura et al. described lymph-node stations 12a, 11p, and 14v (now regarded as D3) as difficult lymph-node stations in laparoscopic D2 lymphadenectomy [33]. They provided detailed description of their approach towards dissection of these stations, and their laparoscopic results were comparable to open lymphadenectomy. These different outcomes may also be due to reporting problems. In the (open surgery) Dutch Gastric Cancer trial, lymph-node station 5 was the most frequent site of non-compliance. This is not a difficult station to dissect, so these findings were attributed to inadequate numbering. The problem of differences in reporting is also reflected in the widely dissimilar findings regarding adherence to D2 lymphadenectomy in the trials by Hu and Park [21, 23]. Both trials reported compliance to D2 lymphadenectomy; in the trial by Hu, > 99% compliance was reported in both open and laparoscopic groups. In the trial by Park et al., adherence to D2 lymphadenectomy was the primary outcome measure; they reported 47 and 43% non-compliance in the laparoscopic and open groups, respectively. Such differences can only be explained by different methods of measuring and reporting compliance to D2 lymphadenectomy. This proves that adequate assessment of adherence to D2 lymphadenectomy is difficult. It should include photo and video documentation of the operative fields, as is required by most recent multicenter trials. Furthermore, all lymph-node stations should be marked or separated from the specimen in the operating room, by the surgeon. Last but not least, the pathologist should be involved actively in the process of identification of separate lymph-node stations. It is known that, in colorectal surgery, the individual pathologist can make a difference in the number of harvested lymph nodes [34].

Probably most widely regarded as the most difficult part of D2 lymphadenectomy (in total gastrectomy) is the splenic hilar lymphadenectomy (station 10). Hosogi and colleagues summarized literature with the results of laparoscopic splenic hilar lymphadenectomy [35]. The mean number of harvested nodes in this area varied from 0 to 5. The authors concluded that, in experienced hands, laparoscopic splenic hilar lymphadenectomy is feasible, but that it cannot be standard of care, due to its technical difficulty. Recent reports suggest that the station 10 and 11 lymph nodes posterior to the splenic vessels need not to be dissected [36, 37]. Too extensive lymphadenectomy may also increase the risk of bleeding complications [38].

Although laparoscopic lymphadenectomy is regarded as more difficult than open, laparoscopy also has advantages. Due to the magnified and detailed view of the present-day laparoscopic systems, lymph nodes can be identified with great accuracy, which may lead to more precise and meticulous lymph-node dissection. Blood loss can be minimized due to rapid identification and controlling of small bleeding. Widely proven and accepted advantages of laparoscopy include less blood loss, less wound complications, and a shorter time to recovery. These advantages may also lead to shorter time intervals between surgery and adjuvant chemotherapy [39]. Adherence to D2 lymphadenectomy itself is again a surrogate marker, namely of survival. A similar adherence to D2 lymphadenectomy in laparoscopy may have the additional benefit that more patients will receive their adjuvant chemotherapy due to less surgical morbidity and faster recovery, and so, overall survival may benefit even more from a laparoscopic approach.

In conclusion, there is no conclusive evidence from randomized trials regarding adherence to D2 lymphadenectomy in gastrectomy, but most trials report no difference in lymph-node harvest. Conclusive evidence regarding dissection results of separate lymph-node stations is not available. The only trial with compliance to D2 lymphadenectomy as primary outcome measure that was published so far found no difference between the laparoscopic and open approach. Evidence regarding D2 lymphadenectomy in laparoscopic total gastrectomy is scarce, and there are no data about lymphadenectomy after neoadjuvant chemotherapy. Results of several large trials are awaited. However, in the meantime, there is sufficient circumstantial evidence that laparoscopic D2 lymphadenectomy is as adequate as in open surgery, and that the laparoscopic approach can be performed safely in experienced centers.
